# Modeling the potential impact of emerging innovations on achievement of Sustainable Development Goals related to maternal, newborn, and child health

**DOI:** 10.1186/s12962-017-0074-7

**Published:** 2017-07-12

**Authors:** Tara Herrick, Claudia Harner-Jay, Craig Shaffer, Greg Zwisler, Peder Digre, Amie Batson

**Affiliations:** 1 PATH, 2201 Westlake Ave. Suite 200, Seattle, WA USA; 2 Applied Strategies, 951 Mariners Island Blvd. Suite 400, San Mateo, CA USA

## Abstract

**Background:**

Innovations that improve the affordability, accessibility, or effectiveness of health care played a major role in the Millennium Development Goal achievements and will be critical for reaching the ambitious new Sustainable Development Goal (SDG) health targets. Mechanisms to identify and prioritize innovations are essential to inform future investment decisions.

**Methods:**

Innovation Countdown 2030 crowdsourced health innovations from around the world and engaged recognized experts to systematically assess their lifesaving potential by 2030. A health impact modeling approach was developed and used to quantify the costs and lives saved for select innovations identified as having great promise for improving maternal, newborn, and child health.

**Results:**

Preventive innovations targeting health conditions with a high mortality burden had the greatest impact in regard to the absolute number of estimated lives saved. The largest projected health impact was for a new tool for small-scale water treatment that automatically chlorinates water to a safe concentration without using electricity or moving parts. An estimated 1.5 million deaths from diarrheal disease among children under five could be prevented by 2030 by scaling up use of this technology. Use of chlorhexidine for umbilical cord care was associated with the second highest number of lives saved.

**Conclusions:**

The results show why a systematic modeling approach that can compare and contrast investment opportunities is important for prioritizing global health innovations. Rigorous impact estimates are needed to allocate limited resources toward the innovations with great potential to advance the SDGs.

**Electronic supplementary material:**

The online version of this article (doi:10.1186/s12962-017-0074-7) contains supplementary material, which is available to authorized users.

## Background

During the past 15 years, innovation has played a tremendous role in global health achievements. For example, innovations in health technologies and systems—including innovations in immunization, malaria prevention and control, nutrition, clean water and sanitation, and education—have contributed to saving the lives of 48 million children since the year 2000 [1].

The Millennium Development Goals (MDGs) showed the world what we can accomplish by galvanizing attention, resources, and accountability to accelerate progress toward a common set of health targets. Yet neonatal, child, and maternal health targets were not met in many low- and middle-income countries.

The recently launched United Nations Sustainable Development Goals (SDGs), the follow-up to the MDGs, outline 17 ambitious goals and related targets for people, planet, and prosperity [2]. Goal 3 for health and well-being has targets ranging from reducing maternal mortality to ensuring “access to quality essential health-care services and access to safe, effective, quality, and affordable essential medicines and vaccines for all.” Fulfilling these goals will require investing in promising new vaccines, drugs, diagnostics, devices, and other innovations, as well as expanding access to existing interventions that have already proven themselves to be safe and effective.

The sources and amounts of financing for global health are expected to change significantly over the next decade. Although development assistance for health grew rapidly between 2000 and 2010 at about 11% per year, annual funding has plateaued at approximately $35 billion in recent years [3]. Domestic funding from low- and middle-income countries to finance their own health expenditures is increasing [4]. In addition, new sources of funding are emerging. The Giving Pledge, for example, represents a group of more than 120 billionaires who have pledged to give at least half of their wealth to philanthropy [5]. In addition, impact investing—or investments that generate social and environmental value as well as a financial return—is expected to increase over the next decade [6]. Although this type of investing is relatively new, some analysts predict it could grow to reach $500 billion [7].

This increasingly complex and diverse funding environment will benefit from new methodologies and tools for making investment decisions. Tools that can incorporate uncertainty and systematically compare and contrast various investment options for social return will be critical to ensure that limited resources are invested effectively.

A few maternal, child, and newborn social impact evaluation modeling tools already exist. The Lives Saved Tool (LiST) developed at Johns Hopkins University, for example, estimates the number of lives that could be saved by increasing coverage of intervention packages for maternal, newborn, and child health (MNCH) [8]. The Maternal and Neonatal Directed Assessment of Technology (MANDATE) model also estimates lives saved for select maternal and neonatal technologies [9].

Innovation Countdown 2030 (IC2030), led by PATH, has crowdsourced, curated, and showcased technologies and interventions with great promise to accelerate progress toward solving the world’s most urgent health challenges [10]. By engaging entrepreneurs, investors, innovators, and experts across sectors and around the world, IC2030 has aimed to raise the visibility of high-potential innovations, thus helping to catalyze investment and accelerate uptake of transformative ideas to improve health and save lives.

IC2030 has used a modeling approach with a different objective than that of either LiST or MANDATE. The approach is especially designed to evaluate how a new innovation could potentially accelerate progress toward specific health targets, in this case an SDG target, and at what cost. The IC2030 team modeled the incremental lives saved and cost of select MNCH innovations between 2015 and 2030. Some of the innovations evaluated in this work cannot be directly modeled with the current versions of LiST or MANDATE. The LiST model focuses exclusively on interventions that directly lead to a coverage change that affects mortality and a limited set of morbidity outcomes. Diagnostic interventions, for example, that improve the timeliness of diagnosis, identify patients in need of various treatments, or improve accuracy of screening are not included. In addition, the MANDATE model does not include child health innovations and costing analysis. Moreover, the countries selected for this analysis—which collectively make up approximately 70% of the global mortality burden for mothers, newborns, and children—are not all represented in the MANDATE tool.

Although initially focused on maternal, newborn and child health, the IC2030 modeling methodology could also be applied to evaluate promising innovations to address HIV, tuberculosis, malaria, noncommunicable diseases, and other disease areas that are critical to achieving SDG health targets. Furthermore, additional innovations to improve MNCH could be evaluated using this framework. This paper examines the IC2030 methodology, results, and insights on innovations that could help to address some of the world’s most urgent health challenges.

## Methods

### Overview

An IC2030 impact model was generated to estimate the incremental lives saved and incremental costs of introducing innovations under development for conditions affecting MNCH. The innovations were crowdsourced from around the world and evaluated and ranked by health area experts as having potential to accelerate progress toward defined SDG health targets. Detailed information on the innovation curation methodology is available at http://ic2030.org/.

In 2015, PATH and Applied Strategies collaborated to model eight innovations, addressing five different health conditions, for the potential to contribute to reaching SDG health targets (Table 1). We modeled prevention, diagnosis, and treatment innovations across a variety of innovation platforms, such as vaccines, drugs, and diagnostics. The modeling methodology was vetted with external advisors, including experts at the Bill & Melinda Gates Foundation and the University of California at San Francisco’s School of Medicine, prior to use. We used a common methodology with consistent parameters for modeling all innovations to allow for comparative analysis. For example, the model time frame, model outputs, and countries included in the analysis are consistent for all innovations unless otherwise noted.

**Table 1:** Summary of modeled innovations

Innovation class	Innovations	Health condition(s)	Treatment continuum	Platform	Target SDG population
New formulations of oxytocin	1) Inhaled oxytocin 2) Sublingual oxytocin	Postpartum hemorrhage (atony)	Prevention and treatment	Drug	Mothers
Uterine balloon tamponade (UBT)	Every Second Matters UBT (ESM-UBT)	Postpartum hemorrhage (atony)	Treatment	Device	Mothers
Simple, safe device for assisted delivery	Odon device^a^	Prolonged and obstructed labor	Treatment	Device	Mothers and neonates
Chlorhexidine for umbilical cord care	1) Chlorhexidine liquid 2) Chlorhexidine gel	Sepsis	Prevention	Drug	Neonates
New treatments for severe diarrhea	1) DiaResQ 2) Qwell	Diarrhea	Treatment	Therapeutics	Children under 5
New tools for small-scale water treatment	Zimba automated batch chlorinator	Diarrhea	Prevention	Device	Children under 5
Portable pulse oximeters to measure oxygen	Non-contact mobile oximeter	Pneumonia	Diagnosis	Device	Children under 5
Better respiratory rate monitors	INSPIRE respiratory rate monitor	Pneumonia	Diagnosis	Device	Children under 5

Source: IC2030 project team

^a^Although the Odon device was modeled for use only during prolonged and obstructed labor, this device may help to reduce morbidity and mortality in other use cases

**Fig. 1:**
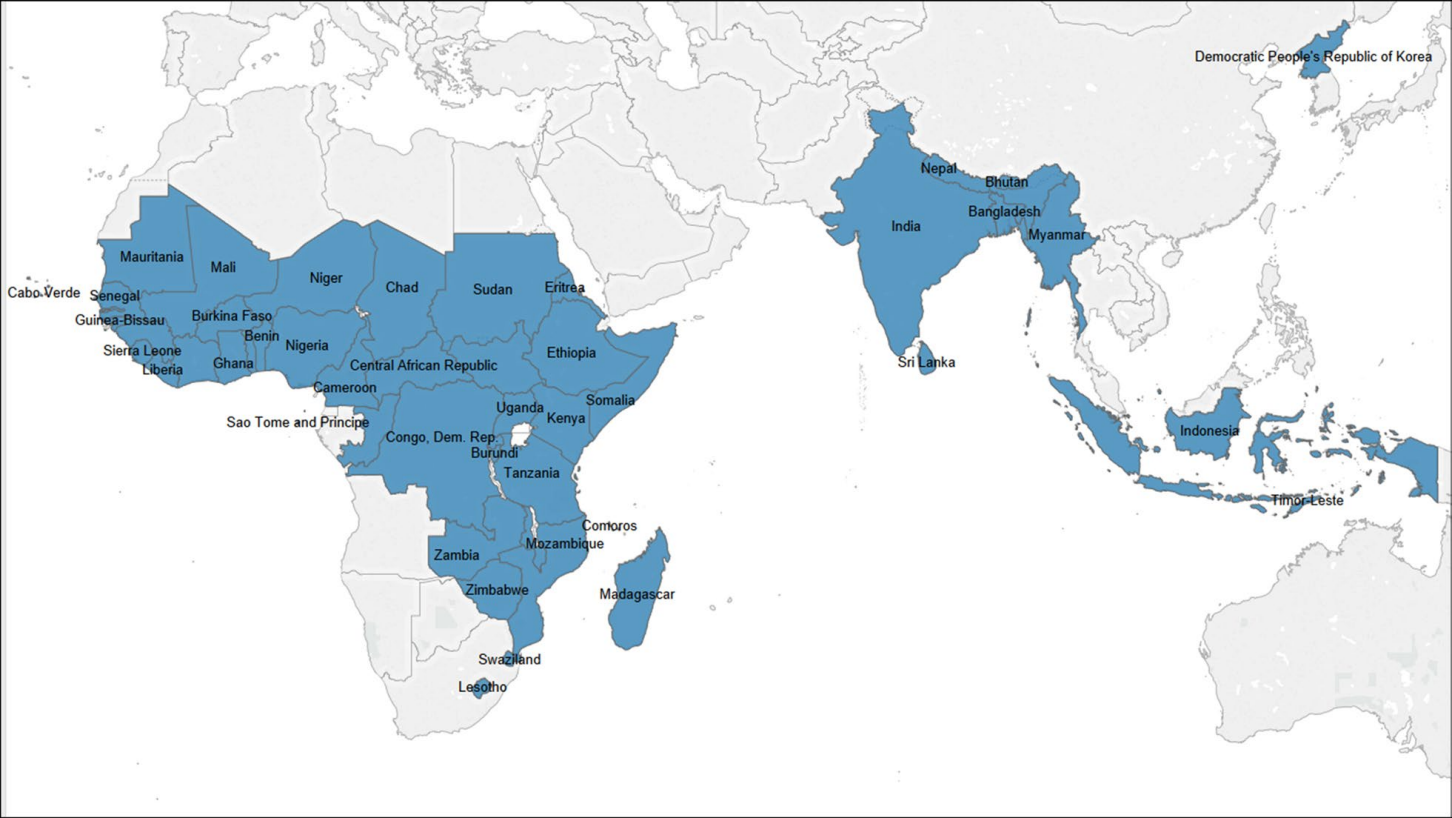
Countries used for modeling impact of health innovations. Source: IC2030 project team. The chlorhexidine model has a slightly different country list based on the World Health Organization’s 2013 recommendations on the postnatal care of the mother and newborn. These guidelines recommend use of chlorhexidine for newborns who are born in the home setting with a neonatal mortality rate of 30 or more deaths per 1000 live births. The percent difference in the number of live births between the chlorhexidine-modeled countries and the other countries is less than 5%. Countries modeled for chlorhexidine use included Angola, Botswana, Equatorial Guinea, Gabon, Afghanistan, and Djibouti. Countries not modeled for chlorhexidine use included Bhutan, Indonesia, North Korea, and Sri Lanka

The IC2030 model forecasts the incremental lives saved and incremental costs between 2015 and 2030 for 49 high-burden countries (Fig. 1). We chose lives saved as the output, rather than disability-adjusted life years (DALYs) or other health impact outputs, to be consistent with the SDG health targets. More specifically, we focused on SDG targets 3.1 and 3.2, which aim to reduce the global maternal mortality ratio to less than 70 per 100,000 live births and to end preventable deaths for newborns and children under 5 years of age, respectively [11].

The countries included in the models are nearly all within sub-Saharan Africa and South-East Asia. Collectively, the countries included in the modeling account for approximately 70% of the global mortality burden for mothers, newborns, and children.

In cases where more than one innovation in a class was nominated, we modeled the innovation class rather than a specific product. For example, two new formulations of oxytocin were identified. One is a fast-dissolving tablet that can be placed under the tongue, and the other is a dry powder that can be administered with a simple inhaler. Both aim to expand access to uterotonic drugs to control postpartum hemorrhage. Unlike conventional oxytocin, these new formulations in development do not require refrigeration during transportation and storage and do not require injection [12, 13]. Our modeling results represent the launch of a new formulation of heat-stable, non-injectable oxytocin rather than use of a specific product.

### Model structure

**Fig. 2:**
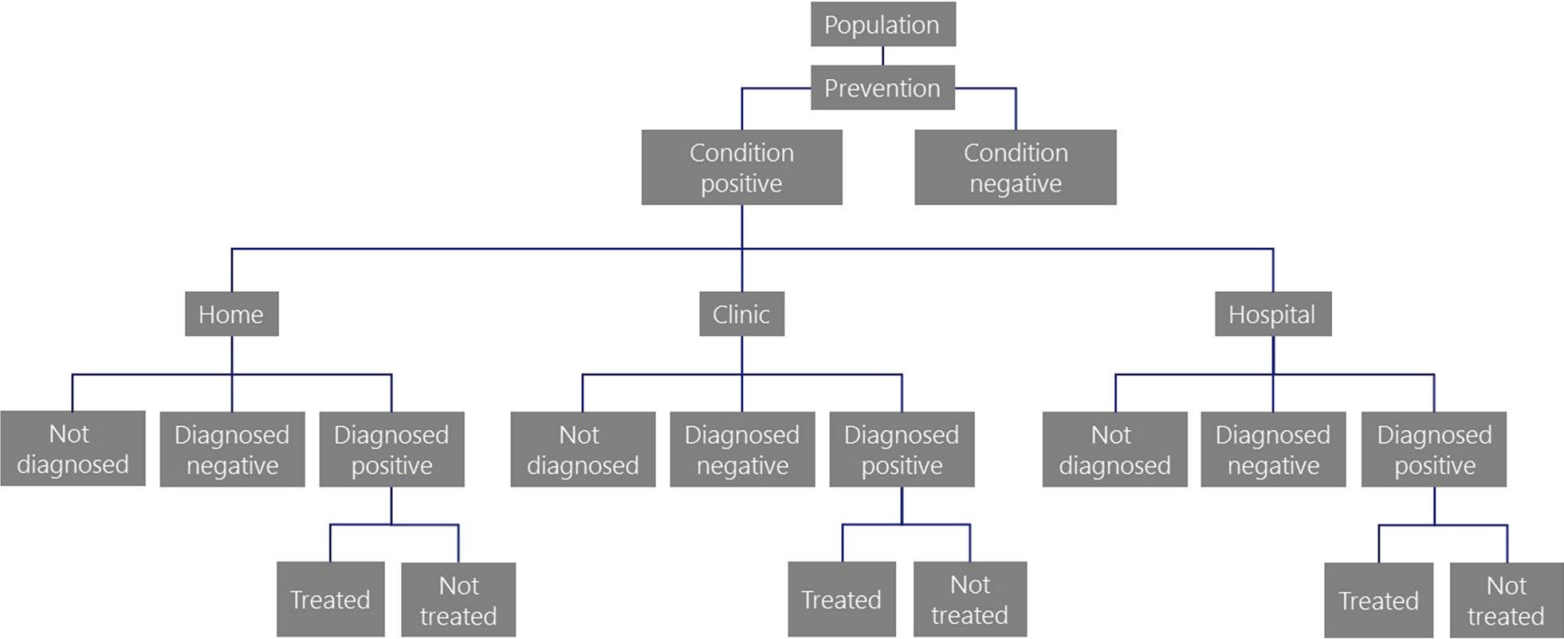
Model structure. Source: IC2030 project team. *Definitions* clinic setting: health posts or health centers with some skilled providers to perform primary care; hospital setting: district, provincial, or regional hospital with skilled providers and surgical capabilities. For patients thought to have a condition based on a false-positive diagnostic test, costs are calculated if treatments occur

We generated an annual country-level, Excel-based forecast model for the 2015 to 2030 time frame for each of five health conditions: postpartum hemorrhage, prolonged and obstructed labor, neonatal sepsis, diarrhea, and pneumonia. Each model starts with an underlying target population size (e.g., pregnant women, neonates, children under five). Epidemiological data are then used to estimate the proportion of the population that is affected by each health condition. Next, the proportion of the population that successfully receives existing prevention, diagnostic, and treatment interventions is calculated based on estimated coverage and effectiveness inputs for home, clinic, and hospital settings (see Fig. 2 for definitions for each setting). Then a case fatality rate is applied for treated and untreated patients to estimate deaths. The model at this stage represents the number of estimated deaths for a specific health condition if no new innovations are introduced. For each health condition model, the 2015 mortality outputs were compared to existing literature to ensure that each model generated a reasonable estimate. Information on data sources is available in the model inputs section.

New prevention, diagnostic, or treatment innovations were then inserted into the appropriate health condition model to estimate the extent to which deaths could be reduced and for what cost. Because each health condition model includes home, clinic, and hospital settings, innovations could be introduced in specific settings that are consistent with their product features. This is particularly important in circumstances where an innovation is substantially easier to use than the status quo intervention. In these cases, it may be possible for the simpler innovation to be administered by less-specialized health care workers who work in lower-level health care facilities. Task shifting to less-specialized health care workers could help to overcome limitations in access to lifesaving solutions due to shortages in the health care workforce. The World Health Organization, in fact, estimates that 83 countries fall below the recommended threshold of 22.8 skilled health providers per 10,000 population, including many countries in Africa and South-East Asia [14]. Figure 2 is a high-level depiction of the model structure. The comparator for each innovation modeled is the current standard of care.

### Model inputs

The IC2030 team identified model inputs in the first quarter of 2015 using available literature and secondary data (see Table 2 for assumptions and sources). Country-level population and epidemiology data were collected from the UN Population Prospects 2012 and relevant disease-specific incidence literature (see Additional file 1) [15]. In cases where the literature reported multiple epidemiological values, the team consulted experts to identify the best estimate when sources disagreed. In addition, we used the most current Demographic and Health Surveys (DHS) estimates for information related to care-seeking behavior (e.g., delivery setting).

**Table 2:** Key innovation model assumptions

Innovation	Launch year	Cost (USD)	Effectiveness			Peak coverage			Notes^a^
			Home	Clinic	Hospital	Home	Clinic	Hospital	Notes
New formulations of oxytocin	2022	$0.50	40%	45%	45%	25%	20%	10%	1) Coverage assumes that new formulations will expand the uterotonic drug market but not replace conventional oxytocin 2) Effectiveness is the same as conventional oxytocin 3) Cost is assumed to be similar to misoprostol
Uterine balloon tamponade	2015	$5.0	N/A	85%	85%	N/A	50%	75%	1) Coverage assumes that new low-cost UBTs will be available for all women who fail uterotonic drugs in clinic and hospital settings 2) Effectiveness is the same as existing approved UBT medical devices 3) Cost is for the ESM UBT product
Simple, safe device for assisted delivery	2018	$25	N/A	72%	81%	N/A	40%	N/A	1) Coverage assumes that the Odon device will expand coverage of existing assisted vaginal delivery devices in clinic settings 2) Effectiveness is similar to existing assisted vaginal delivery devices 3) Cost is similar to other instrumented delivery options
Chlorhexidine for umbilical cord care	2015	$0.36	23% reduction in neonatal mortality	N/A	N/A	55%	N/A	N/A	1) Coverage assumes that chlorhexidine is available to women who give birth in the home setting 2) Effectiveness is based on meta-analysis data in low resource countries 3) Cost is based on the UNICEF supply catalog price
New treatments for severe diarrhea	2018	$.50	Improves ORS coverage and adherence	N/A	N/A	80%	N/A	N/A	1) Will be used concurrently with ORS and increase coverage of ORS by 15 percentage points 2) Not effective at reducing mortality in isolation. The product will help expand access and improve adherence to ORS 3) Cost is based on ORS
New tools for small-scale water treatment	2015	$70	84%	N/A	N/A	25%	N/A	N/A	1) Coverage estimates apply to the proportion of the population that receives water through community sources and are based on coverage of home water treatment solutions 2) Effectiveness based on data for the Safe Water System (reduction in diarrhea incidence) 3) Cost is based on similar products; product will serve 1000 people
Portable pulse oximeters to measure oxygen	2015	$40	N/A	85%	85%	N/A	70%	80%	1) Coverage based on estimates for other durable medical equipment (e.g. blood pressure measurement) in low resource settings 2) Effectiveness reported is for detecting oxygen saturation 3) Cost based on similar medical devices
Better respiratory rate monitors	2015	$50	N/A	80%	80%	N/A	50%	60%	1) Coverage based on other estimates for durable equipment (blood pressure measurement) in low resource settings 2) Effectiveness is based on publications evaluating respiratory rate counting and chest wall indrawing to diagnose pneumonia 3) Cost is based on similar medical devices

Source: IC2030 project team

*Definitions* coverage: the availability and use of an innovation in a given setting; effectiveness: the efficacy of an innovation adjusted for real-world settings (by contrast, efficacy is the outcome of an innovation under ideal conditions); home setting: outside a health care facility with limited or no access to skilled health care providers

^a^Assumptions related to effectiveness were based on available published evidence. For example, the effectiveness of the uterine balloon tamponade was based on data from [22]. A list of key sources for effectiveness assumptions is available from the corresponding author

Secondary information was also reviewed and collected on the coverage and effectiveness of existing preventative, diagnostic, and treatment interventions in both the Africa and South-East Asia regions for the home, clinic, and hospital settings for each health condition. The inputs on effectiveness in the model are assumed to be the same as published data for the existing comparator technology in cases where a comparator technology is available. For example, new formulations of oxytocin are assumed to have the same effectiveness as existing formulations of oxytocin. In cases where a comparator technology is not available (e.g., chlorhexidine), clinical trial data were used as the input for effectiveness.

Publicly available information was reviewed for each innovation to determine model assumptions for launch timing, peak coverage rate and effectiveness (see Table 2). For all products, we assumed a 5-year linear time to peak coverage.

## Results

**Fig. 3:**
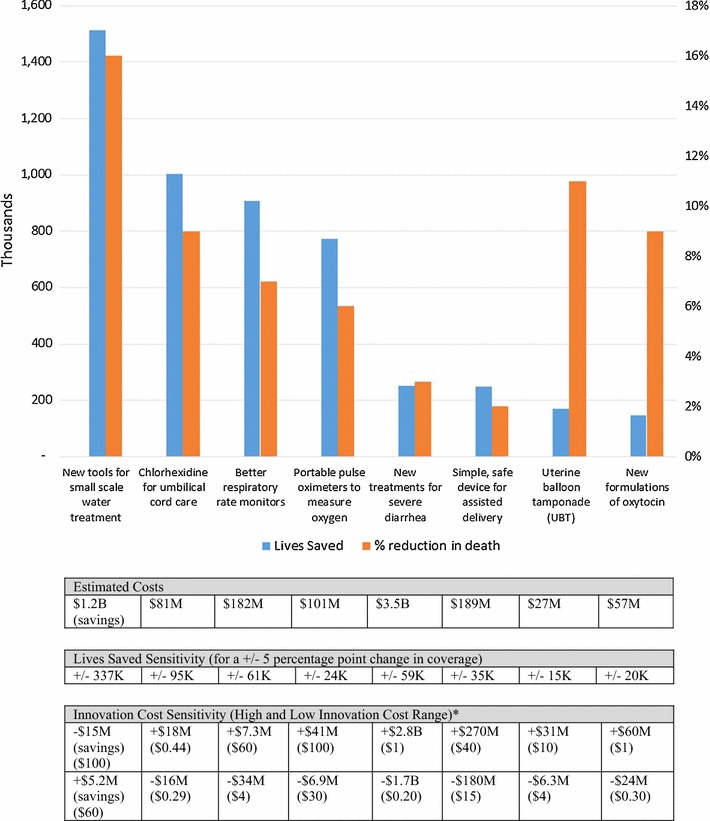
Modeled impact of eight innovations under development. Source: IC2030 project team. *Upper and lower costs modeled are indicated in parentheses. Costing information is specific to the innovation cost, introduction cost, downstream treatment costs, and potential treatment costs that could be averted. Research and development costs and costs related to economic productivity are not included. The sensitivity analysis shows how the estimates for lives saved would change if coverage was adjusted plus or minus 5 percentage points

The largest projected health impact in this study was for a new tool for small-scale water treatment that automatically chlorinates water to a safe concentration without using electricity or moving parts [16, 17]. An estimated 1.5 million deaths from diarrheal disease among children under the age of five could be prevented between now and 2030 (Fig. 3). In addition, this was the only innovation modeled for which we found a cost savings (estimated $1.2 billion). The large number of diarrhea episodes per child less than 5 years old per year (estimated 2.4 in South-East Asia and 3.3 in sub-Saharan Africa) and the large number of treatments used to manage diarrhea (e.g., ORS, antibiotics, and zinc) result in high treatment costs. Reducing the incidence of diarrhea reduces the number of treatments and results in large cost savings.

Chlorhexidine for umbilical cord care was associated with the second highest number of lives saved (estimated 1.0 million). Like the tool for small-scale water treatment, this innovation prevents the onset of disease. Unsanitary conditions during childbirth and a lack of antiseptics in the first week after birth increase the risk of sepsis. Using chlorhexidine for births that occur in the home setting dramatically reduces the risk of severe infection. Although cost savings were not found for this innovation, the cost for chlorhexidine is estimated at only $0.36 through the UNICEF Supply Division Catalogue [18].

To understand the robustness of the model outputs, sensitivity analysis was performed on the following key innovation inputs: coverage, innovation cost, and effectiveness. Each input was varied in isolation, while keeping the other inputs at baseline. The sensitivity analysis for the coverage parameter highlights the importance of understanding adoption of global health innovations. Adjusting this value by 5 percentage points in either direction results in changing the number of lives saved by plus or minus 337,000 for new tools for small-scale water treatment and 95,000 for chlorhexidine. Changing the effectiveness by 5 percentage points had a 10% change or less in the number of lives saved for all innovations except the Odon device, which had a 14% change (data not shown). Lastly, we put ranges around the innovation cost to understand how the modeled costs change. The modeled costs in this analysis include the innovation cost, introduction costs, downstream treatment costs, and potential treatment costs that could be averted. Adjusting the innovation cost for the small-scale water treatment had a very small change in the modeled costs. This is likely due to the large number of diarrheal disease treatments that occur. Other innovations like the new formulations for oxytocin, the uterine balloon tamponade (UBT), the Odon device, and new treatments for severe diarrhea were more sensitive to innovation cost changes. It should be noted that within the sensitivity analysis, price does not influence coverage. Figure 3 summarizes the modeling results and sensitivity analysis for all eight innovations.

## Discussion

Overall, the modeling results showed that preventative innovations targeting health conditions with high mortality burden had the greatest impact in terms of the absolute number of estimated lives saved. Not surprisingly, innovations that target conditions with a substantial mortality burden have more potential to save lives than innovations that target conditions with a more modest mortality burden. Preventative innovations likely contributed to a larger health impact than diagnostic or treatment innovations because there are fewer dependencies in the continuum of care. However, because preventative innovations are used in broader populations that do not yet have disease, costs need to be carefully monitored.

Treatment innovations, in contrast, require that a patient be diagnosed prior to receiving treatment. In some cases, the coverage of the existing diagnostic may be low, or the existing diagnostic may have low sensitivity and specificity. These diagnostic inefficiencies limit the opportunity for a new treatment to have impact because fewer individuals are eligible for treatment. However, if these diagnostic inefficiencies are addressed, there are additional opportunities and value for the new treatment or the existing treatment intervention.

Structuring the model to represent different levels of the health system (home, clinic, and hospital settings) provided useful information. Different introduction setting scenarios were explored for the innovations to gain insight on the settings that could have the most impact. While many different scenarios can be examined using this model structure, the results in this paper represent introduction setting scenarios that are consistent with each innovation’s product features. The UBT, for example, showed an 11% reduction in maternal mortality due to postpartum hemorrhage if introduced exclusively in clinics and hospitals. At present, there is a gap in the treatment continuum for women who suffer from refractory bleeding due to postpartum hemorrhage caused by atonic uterus in all settings. Introducing the UBT in clinics and hospitals, staffed by skilled birth attendants, is a logical first step. However, expanding use to the home setting would augment impact if in the future non-skilled providers could administer the product. This scenario was not modeled because existing studies have focused on demonstrating that UBTs are safe and effective when administered by trained health care providers in health care facilities. Other innovations, such as new formulations of oxytocin to prevent and treat postpartum hemorrhage, will have more impact by expanding use to the home setting. This is because oxytocin is already available in many clinics and facilities in high-burden countries.

The model we generated can evaluate multiple innovations simultaneously within a specific health condition. However, we sought to understand how each innovation could contribute to the SDG health targets in isolation. Results within a specific health category should not be added together.

Our analysis had several limitations. The project team used historical information for the underlying inputs in the model, and there is no guarantee that these trends will continue throughout the forecast time frame. In addition, all of the modeled innovations were still undergoing research and development when modeling occurred. For this exercise, we assumed that each innovation would reach its technical and regulatory milestones and launch. However, there is substantial technical and regulatory risk for all health care innovations, and there is no guarantee that these innovations will actually become available in the countries included in this analysis.

There is also significant uncertainty in the coverage assumptions for each of the modeled innovations. We did not conduct primary market research for this analysis or consult with manufacturers. In addition, the costing information included in this analysis is specific to the innovation cost, introduction costs, downstream treatment costs, and potential treatment costs that could be averted. Research and development costs and costs related to economic productivity are not included. Although data were available for the effectiveness of most innovations in low-resource countries (see Table 2), country-level variation in effectiveness was not considered.

Information obtained from these models is aimed at supporting comparative analysis of the potential impact that innovations could have toward the SDG health targets. The information should be viewed as estimates, and inputs should be updated as new information becomes available.

## Conclusions

This analysis estimates how promising innovations in development can contribute toward SDG health targets 3.1 and 3.2. The modeling methodology uses a standardized approach that enables comparative analysis across innovations. It uses sensitivity analysis to show the uncertainty surrounding the peak innovation coverage assumption to understand how outputs change given different input values. To our knowledge, this approach is the first work of its kind that evaluates how innovations can contribute toward achieving the SDG health targets.

There is an opportunity to expand and enhance this modeling methodology to other innovations outside of the MNCH areas. The SDG health targets focus on “ensuring healthy lives and promoting well-being for all at all ages.” The goals include specific health targets for AIDS, tuberculosis, malaria, neglected tropical diseases, noncommunicable diseases, family planning, substance abuse, and injury. By identifying promising innovations to accelerate progress toward these goals now, donors, governments, policymakers, health care providers, technology developers, and other key global health stakeholders can focus efforts on the solutions with the most potential to transform health and maximize resources.

About one in five innovations nominated for the IC2030 project were crosscutting. These health system and platform innovations, including digital tools and broad diagnostics, have the potential to address multiple SDG health targets simultaneously. Modeling crosscutting innovations for impact is more challenging because it requires evaluating multiple health outcomes. However, future modeling iterations should strive to incorporate these innovations because of their strong potential to accelerate progress toward multiple SDG health targets.

As we approach 2030, it will be important to continue to revise and refine the model inputs to generate forecast estimates that reflect the most current thinking. There is a need to improve data collection on the availability of health care interventions in low-resource settings to understand how needs are changing over time. Health care data collection vendors such as IMS Health have a limited reach in low-resource markets, likely because demand for this type of data is still nascent. Recent publications aiming to describe the coverage of specific drugs in global markets using IMS Health data mention data gaps in many of the high-burden countries featured in this analysis [19, 20]. Although some publicly available data resources exist—including DHS, Multiple Indicator Cluster Surveys, the international drug price indicator guide, and peer-reviewed publications—many of these sources are infrequently updated and are limited in terms of the scope in what they collect.

There is an opportunity and a need to improve data collection in low-resource settings, and some funders are beginning to support more work in this area [21]. The Bill & Melinda Gates Foundation, for example, awarded six $100,000 grants in 2013, through its Grand Challenges Explorations program, that are focused on better data collection. In addition, the foundation also awarded PATH a grant to improve the collection, quality, and use of immunization data. These investments in better data collection will continue to improve the accuracy of health impact forecasting.

Health impact modeling tools are critical for ensuring that limited resources are spent on the most promising global health opportunities. The LiST and MANDATE tools are helpful resources for estimating lives saved for select interventions. However, at present, the online tools do not have the capability to estimate the impact of all of the innovations considered in this assessment. This work highlights the importance of using a systematic approach to compare and contrast opportunities to accelerate progress toward reaching the SDG health targets. Continued investments in data collection will improve the accuracy of health impact modeling tools between now and 2030. Refining the existing health impact models generated in this work and expanding the analysis to other health areas will help to accelerate progress toward solving the world’s most urgent health issues.

## Additional file

**Additional file 1.** Sources on epidemiologic data.

## Abbreviations

DALY: disability-adjusted life year
DHS: Demographic and Health Surveys
ESM-UBT: Every Second Matters UBT
IC2030: Innovation Countdown 2030
LiST: Lives Saved Tool
MANDATE: Maternal and Neonatal Directed Assessment of Technology
MDGs: Millennium Development Goals
MNCH: maternal, newborn, and child health
ORS: oral rehydration solution
SDGs: Sustainable Development Goals
UBT: uterine balloon tamponade
