# Supplementary material for: Modeling the potential impact of emerging innovations on achievement of Sustainable Development Goals related to maternal, newborn, and child health
Source: Cost Eff Resour Alloc. 2017 Jul 12;15:12. doi: 10.1186/s12962-017-0074-7 (PMC5506623; doi:10.1186/s12962-017-0074-7)
Supplement: Supplementary file 1 — Additional file 1. Sources on epidemiologic data. [file 12962_2017_74_MOESM1_ESM.docx]

| **Innovation Class** | **Intervention** | **Health Conditions(s)** | **Treatment continuum** | **Source on epidemiologic data** |
| --- | --- | --- | --- | --- |
| New formulations of oxytocin | 1. Inhaled oxytocin 2. Sublingual oxytocin | Postpartum hemorrhage (atony) | Prevention and treatment | Carroli G, Cuesta C, Abalos E, Gülmezoglu AM. Epidemiology of postpartum haemorrhage: a systematic review. Best Pract Res Clin Obstet Gynaecol 2008, 22:999–1012.  Abrams ET, Rutherford JN. Framing postpartum hemorrhage as a consequence of human placental biology: an evolutionary and comparative perspective. Am Anthropol 2011, 113:417–430. |
| Uterine balloon tamponade (UBT) | Every Second Matters UBT (ESM-UBT) |  | Treatment |  |
| Simple, safe device for assisted delivery | Odon device | Prolonged and obstructed labor | Treatment | Dolea C, AbouZahr C. Global burden of obstructed labour in the year 2000. Global Burden of Disease 2000. Geneva: World Health Organization; 2003.  Randhawa I, Gupta KB, Kanwal M. A study of prolonged labour. J Indian Med Assoc 1991, 89:161–163.   Kaye DK, Kakaire O, Osinde MO. Systematic review of the magnitude and case fatality ratio for severe maternal morbidity in sub-Saharan Africa between 1995 and 2010. BMC Pregnancy and Childbirth 2011, 11:65. |
| Chlorhexidine for umbilical cord card | 1. Chlorhexidine liquid 2. Chlorhexidine gel | Sepsis | Prevention | World Population Prospects. United Nations Population Division, 2012. <https://esa.un.org/unpd/wpp/>.  RTI International. MANDATE Model, Version 1.1.81., Data Version 1.1.122. http:// <http://mandate4mnh.org/>. |
| New treatments for severe diarrhea | DiaResQ | Diarrhea | Treatment | Fischer Walker CL, Perin J, Aryee MJ, Boschi-Pinto C, Black RE. Diarrhea incidence in low- and middle-income countries in 1990 and 2010: a systematic review. BMC Public Health 2012, 12:220.  Bardhan P, Faruque ASG, Naheed A, Sack DA. Decrease in shigellosis-related deaths without Shigella spp.-specific interventions, Asia. Emerging Infect Dis 2010, 16:1718–1723.  Bhandari N, Bhan MK, Sazawal S. Mortality associated with acute watery diarrhea, dysentery and persistent diarrhea in rural North India. Acta Paediatr 1992, 81 Suppl 381:3–6.  Robberstad B, Strand T, Black RE, Sommerfelt H. Cost-effectiveness of zinc as adjunct therapy for acute childhood diarrhoea in developing countries. Bulletin of the World Health Organization 2004, 82:523–531.  Bennish ML, Wojtyniak BJ. Mortality due to shigellosis: community and hospital data. Rev Infect Dis 1991, 13 Suppl 4:S245–51.  Case Fatality Rates for untreated non-dysentery diarrhea (Calculated CFR based on published diarrhea incidence and deaths data retrieved from 2004 WHO Global Burden of Disease. |
| New tools for small-scale water treatment | Zimba automated batch chlorinator |  | Prevention |  |
| Portable pulse oximeters to measure oxygen | Non-contact mobile oximeter | Pneumonia | Diagnosis | O'Brien KL, Wolfson LJ, Watt JP, Henkle E, Deloria-Knoll M, McCall N, Lee E, Mulholland K, Levine OS, Cherian T, Hib and Pneumococcal Global Burden of Disease Study Team. Burden of disease caused by Streptococcus pneumoniae in children younger than 5 years: global estimates. The Lancet 2009, 374:893–902.  Rudan I, O'Brien KL, Nair H, Liu L, Theodoratou E, Qazi S, Lukšić I, Fischer Walker CL, Black RE, Campbell H, Child Health Epidemiology Reference Group (CHERG). Epidemiology and etiology of childhood pneumonia in 2010: estimates of incidence, severe morbidity, mortality, underlying risk factors and causative pathogens for 192 countries. J Glob Health 2013, 3:010401.  Tiewsoh K, Lodha R, Pandey RM, Broor S, Kalaivani M, Kabra SK. Factors determining the outcome of children hospitalized with severe pneumonia. BMC Pediatr 2009, 9:15. |
| Better respiratory rate monitors | INSPIRE Respiratory Rate Monitor |  |  |  |
